# Acute disseminated encephalomyelitis with delayed onset and feasibility of the Miethke shunt and sensor reservoir system: a case report

**DOI:** 10.1007/s00381-021-05188-7

**Published:** 2021-06-16

**Authors:** Gunnar Liminga, Anna Grabowska, Dýrleif Pétursdóttir, Kristina G. Cesarini, Elham Rostami, Christoffer Ehrstedt

**Affiliations:** 1 Department of Women’s and Children’s Health, Uppsala University , Uppsala, Sweden; 2 Department of Surgical Sciences, Radiology, Uppsala University, Uppsala, Sweden; 3 Department of Neuroscience, Ophthalmology, Uppsala University, Uppsala, Sweden; 4 Department of Neuroscience, Neurosurgery, Uppsala University, Uppsala, Sweden; 5 Department of Neuroscience, Karolinska Institute, Stockholm, Sweden

**Keywords:** Acute demyelinated encephalomyelitis, Delayed onset, High intracranial pressure, Ventriculoperitoneal shunt, Sensor reservoir

## Abstract

Acute disseminated encephalomyelitis (ADEM) is an immune-mediated demyelinating central nervous system disorder with predilection for early childhood. Delayed onset of ADEM is rare, and herein we present a previously healthy 5-year-old boy, with an unusual clinical course of ADEM with high intracranial pressure (ICP) and acute visual loss that was at first diagnosed as idiopathic intracranial hypertension without papilledema (IIHWOP). The boy underwent acute neurosurgical intervention with ventriculoperitoneal (VP) shunt using Miethke valve and sensor reservoir system and received high-dose steroid treatment with symptom relieve within days. This is the first case report using this system in such a young child, and we find it feasible and valuable also in younger children when VP shunt with ICP measurement is indicated.

## Introduction

Acute disseminated encephalomyelitis (ADEM) is an immune-mediated demyelinating central nervous system disorder with predilection for early childhood. Common presenting symptoms are encephalopathy, fever, headache, nausea, vomiting, and ataxia. Diagnosis is made by a combination of clinical and magnetic resonance imaging (MRI) findings of the brain and medulla, and provided other etiologies of inflammatory and infectious causes have been excluded [1, 2].

Herein, we present a previously healthy 5-year-old boy, with an unusual clinical course of ADEM, eventually causing a very high intracranial pressure (ICP) resulting in an acute neurosurgical intervention with ventriculoperitoneal (VP) shunt.

## Case presentation

**Fig. 1:**
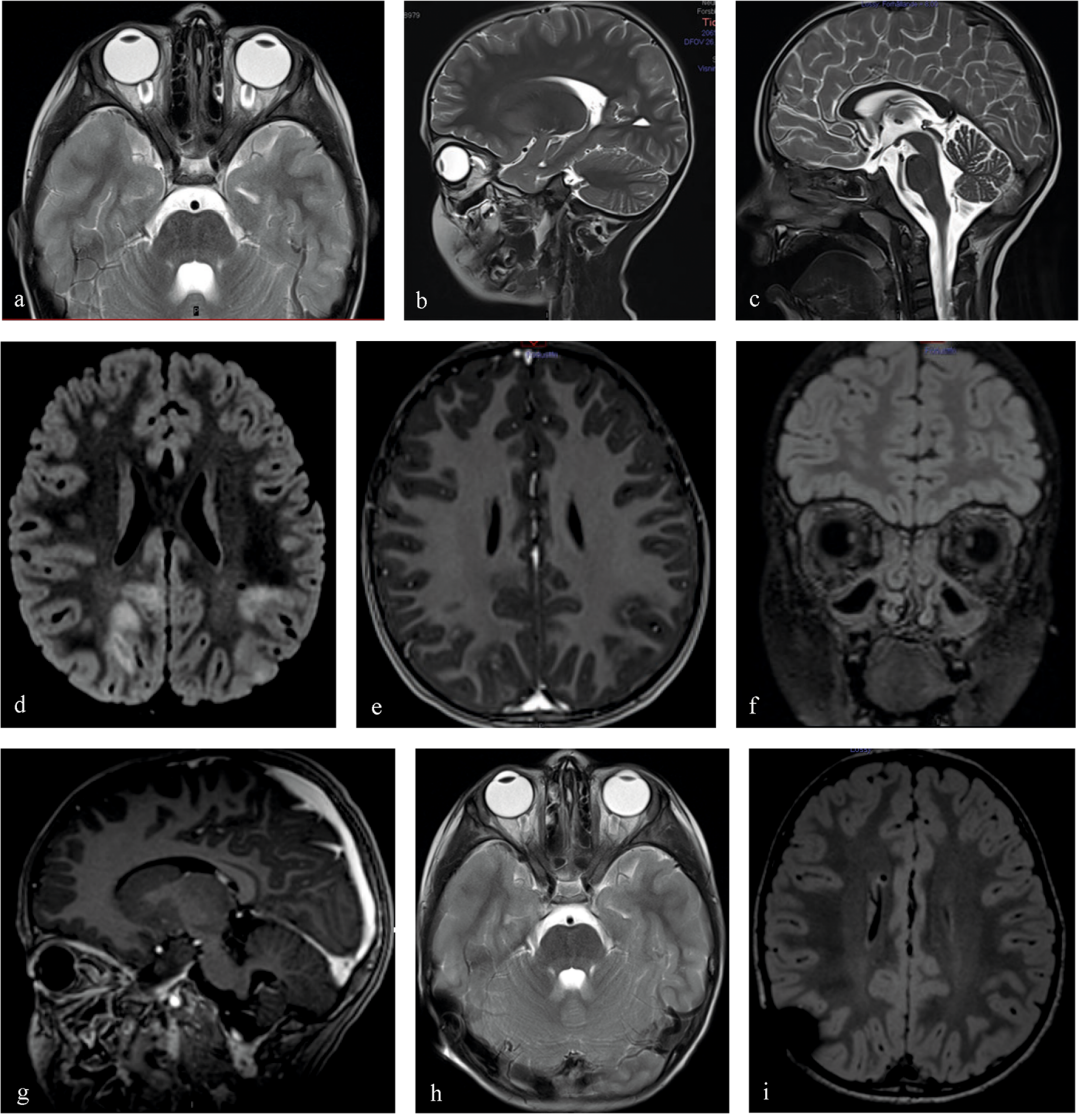
**a**–**c** T2-weighted sequences from the initial MRI of the brain, performed during investigation of the headache and nausea, demonstrate findings consistent with IIH. **a** Transverse T2-weighted image shows significant distention of the perioptic nerve sheaths with flattening of the posterior sclera of both globes; **b** sagittal T2-weighted image showing same findings and in addition vertical tortuosity of the intraorbital optic nerve; **c** sagittal T2-weighted image with mild reduction in the height of the hypophysis. **d**–**g** MRI sequences obtained at the time of rapid deterioration with impairment of vision and encephalopathy. **d** Transverse FLAIR image demonstrates multiple bilateral subcortical hyperintense lesions dominating in the parietal and occipital lobes; **e** transverse T1 gadolinium (Gd) image shows incomplete ring or nodular contrast enhancement in some of the subcortical lesions; **f** coronal FLAIR image shows bilateral optic nerve head protrusion into the globes; **g** sagittal T1 Gd image showing protrusion of the optic nerve head into the globes and diffuse contrast enhancement in both optic nerves. **h**–**i**: Follow-up MRI 2 months after VP shunt placement and steroid treatment. **h** T2 transverse image shows regress of distention of perioptic nerve sheaths, posterior globe flattening, and optic nerve head protrusion into the globe; **i** FLAIR transverse image shows complete regression of the intracerebral signal changes

Two months prior to symptom debut, the boy had a flu-like episode. Medical advice was sought at the local county hospital after 3 weeks of increasing symptoms with headache and nausea. Routine blood sampling and neurologic and ophthalmologic examinations were normal. Magnetic resonance imaging revealed optic nerve sheath distension and a minor bilateral intraocular protrusion of the optic nerves (Fig. 1a–c). A lumbar puncture (LP) demonstrated an elevated opening pressure of 39 cm H_2_O and slightly elevated white cell count (2 poly, 8 mono) in the cerebrospinal fluid (CSF). He was diagnosed with idiopathic intracranial hypertension without papilledema (IIHWOP), and treatment with acetazolamide (25 mg/kg/day) was initiated.

One month after initiation of acetazolamide treatment, his medical condition deteriorated rapidly. He became encephalopathic within 24 h and experienced worsening of headache, nausea, and impaired vision. Ophthalmologic assessment revealed a visual acuity of 0.1 decimal in the right eye and only ability for light perception in the left eye, with a stage 5 papilledema bilaterally. Lumbar puncture demonstrated an opening pressure of > 50 cm H_2_O. An acute CT scan showed signs of high ICP. At this stage, he was transferred to Uppsala University Hospital, a tertiary referral center for children and adolescents in need of neurosurgical and pediatric neurology care and treatment.

**Fig. 2:**
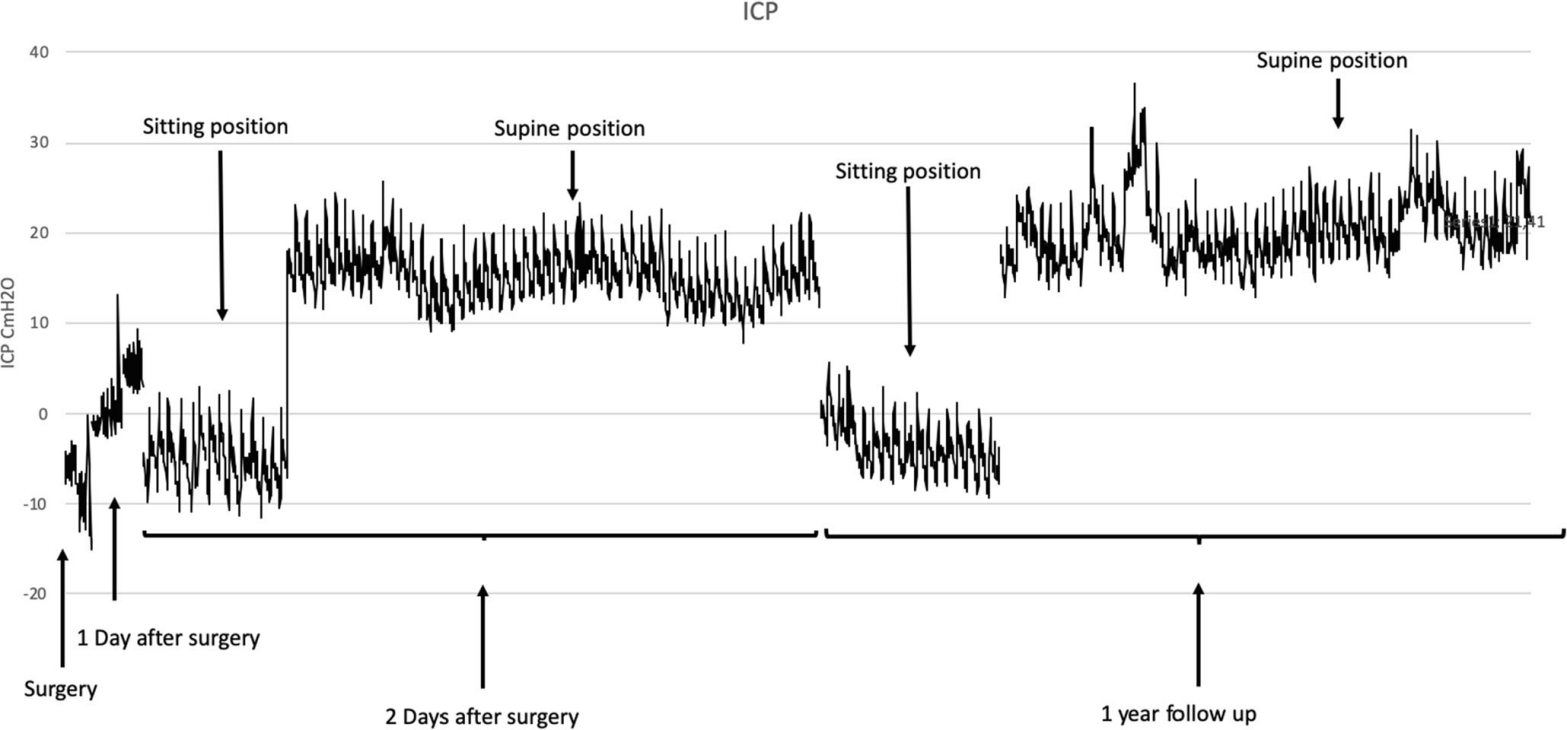
Timeline of ICP measurements with the Miethke sensor system following surgery. ICP values of −10 up to +5 cm H_2_O was noted directly postoperatively. Daily ICP measurements postoperatively demonstrated values of −10 to 0 cm H_2_O in sitting position and +10 to 20 cm H_2_O in supine position. Measurements were performed during changes in patient position in order to assess shunt valve function and suitable settings. Clinical symptoms and ICP measurements together guided the valve settings

An acute MRI revealed extensive signs of neuroinflammation (Fig. 1d−g). A new diagnostic work-up was performed (Table 1), and a diagnosis of ADEM with bilateral optic neuritis (ON) causing high ICP and visual loss was made. Due to the severe situation with an eminent threat of losing visual function, he received a VP shunt within hours from arrival to the pediatric intensive care unit. An ICP sensor reservoir/prechamber system (Christoph Miethke, Potsdam, Germany) was connected to a Miethke, proGAV 2.0 valve, with opening pressure set at 18 cm H_2_O and proSA, shunt assistant, setting of 10 cm H_2_O. The ICP sensor reservoir enabled daily ICP measurements postoperatively and demonstrated normal values (Fig. 2). Due to the MRI findings, a 5-day treatment with high-dose IV methylprednisolone (30mg/kg × 1) was initiated, followed up with 2 weeks of high-dose prednisolone (1mg/kg) with a tapering for an additional 6 weeks.

**Table 1:** A summary of the diagnostic work-up performed when the patient´s medical condition deteriorated

		Normal range
Neuroinflammation (CSF)		
CXCL13	94	< 7.8
NMO-ab (aquaporin4-ab)	Negative	
MOG-ab	Negative	
Anti-neuronal ab	Negative	
CSF		
Glucose	Normal	
Albumin	Normal	
Lactate	Normal	
White blood cells	16 poly, 41 mono	< 6
IgG index	0.78	< 0.63
Oligoclonal bands	Negative	
Infectious		
Toxoplasmosis	Negative	
Meningoencephalitis block*	Negative	
TBE and Lyme	Negative	
Parvo	Negative	
Rubella	Negative	
Other		
CBC	Normal	
Creatinine	Normal	
ASAT, ALAT	Normal	
TFT	Normal	
ACE	Normal	

*NMO* neuromyelitis optica, *MOG* myelin oligodendrocyte glycoprotein, *TBE* tick-borne encephalitis, *CBC* complete blood count, *ASAT* aspartate aminotransferase, *ALAT* alanine aminotransferase, *TFT* thyroid functions tests, *ACE* angiotensin-converting enzyme

*Meningoencephalitis block; *HSV-1* herpes simplex virus type 1, HSV-2, entero-, varicella zoster-, cytomegalo- and Epstein-Barr virus

**Fig. 3:**
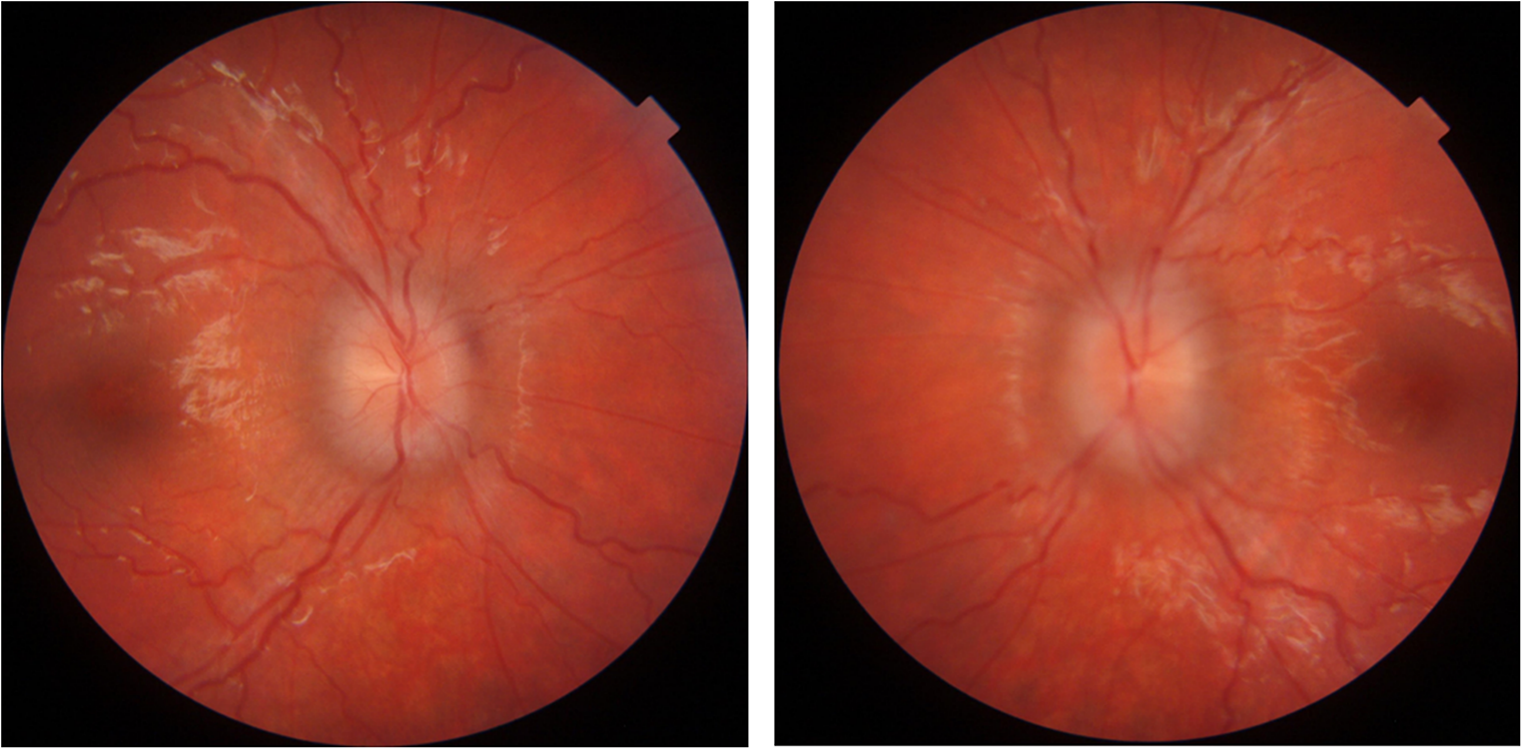
Fundus photography of the patient 6 days after the VP shunt operation

There was a successive alleviation of symptoms. Visual acuity assessed 6 days postoperatively was 0.8 and 0.63 on the right and left eyes, respectively, with regression of papilledema to stage 4 on the right eye and less pronounced stage 5 on the left eye (Fig. 3).

Further clinical and neuroradiological follow-ups (Fig. 1h–i) have been uneventful with the exception of a few episodes with recurrent headache. At 1-year follow-up, there was full recovery with normal visual function and without any neurologic deficits. Multiple ICP measurements with the Miethke sensor system were within normal range, both at episodes with recurrent headache and at 1-year follow-up (Fig. 2).

## Discussion

Here we have presented an atypical case of ADEM with ON initially mimicking IIH/IIHWOP. Somewhat confusing was the initial prolonged clinical course together with normal neuroradiological findings. However, pleocytosis in CSF is not consistent with IIH/IIHWOP, and our case highlights the importance of diagnostic scrutiny in this regard. High levels of CXCL13 and white matter changes on MRI supported the final diagnosis of a demyelinating CNS disorder.

Severe presentations of ADEM resulting in admission to intensive care units have been described [3]; however, alleviation of very high ICP with neurosurgical interventions such as hemicraniectomy or VP shunt are limited to case reports [4, 5]. Timing of neurosurgical intervention may be a meticulous task when a patient presents with signs of increased ICP secondary to neuroinflammatory disease. It is possible that our patient would have responded well to just steroid treatment. However, due to the quick clinical deterioration and ophthalmological findings, the decision was made to relieve the high ICP with a VP shunt and treat the underlying neuroinflammation. One could also argue that an acute but temporary CSF diversion, such as ventriculostomy or lumbar drainage, could have been used. However, in the light of an already protracted clinical course and IIH being a possible differential diagnosis at the time of neurosurgical intervention, a VP shunt was considered the best alternative. Our case supports measuring lumbar puncture opening pressure as a clinical routine in children with ADEM, as suggested by Orbach et al. [6].

This is the first report describing the use of Miethke, proGAV 2.0, proSA, and ICP sensor reservoir/prechamber system in a preschool child. For a few years, Christoph Miethke GmbH has provided a pressure sensor system that allows ICP to be measured without performing lumbar punctures or invasive ICP monitoring in the neurointensive care unit. It also has the advantage of being MRI compatible. Thus, the Miethke shunt valve and sensor reservoir system are highly suitable for complex cases when repeated MRI examinations are expected and/or valve adjustments are predicted to be challenging due to insufficient guidance of neuroimaging findings. The ICP measurement is not dependent on the position of the patient and can be performed in supine, sitting, and standing positions. This is even more beneficial in children when LP is performed for ICP measurement, and they need to be sedated. Furthermore, the ICP sensor represents the intracranial pressure better compared to lumbar measurements. Santes et al. reported successful use of this system in 25 patients including children, youngest 13 years of age, and that ICP-guided valve adjustments positively changed the clinical state in 18 of 25 patients [7].

## Conclusion

Delayed onset of ADEM occasionally occurs. In patients presenting with signs of raised ICP and/or severe visual impairment, there is a clinical rationale for measuring the lumbar opening pressure. In cases when neurosurgical intervention is necessary, it is advisable to consider the possibility of a MR compatible Miethke shunt valve and sensor reservoir system to measure ICP in a non-invasive way, especially in younger children.
